# Prediction nomogram for coronary artery aneurysms at one month in Kawasaki disease

**DOI:** 10.1186/s13052-023-01551-3

**Published:** 2023-11-06

**Authors:** Yunjia Tang, Chuxin Ding, Qiuqin Xu, Wanping Zhou, Yiming Qin, Meihua Lu, Haitao Lv

**Affiliations:** 1 Department of Cardiology, Children’s Hospital of Soochow University, No 92, Zhongnan Street, Suzhou, People’s Republic of China; 2 Department of Pediatrics, Changshu Hospital Affiliated to Nanjing University of Chinese Medicine, No 6, Huanghe Road, Changshu, People’s Republic of China

**Keywords:** Mucocutaneous lymph node syndrome, Coronary artery lesions, Coronary artery aneurysms, Prediction model

## Abstract

**Background:**

Coronary status at one month after Kawasaki disease (KD) onset had a great significance. The present study aimed to establish a prediction model for coronary artery aneurysms (CAA) at one month in children with KD.

**Methods:**

Patients with a diagnosis of KD between May 2017 and Dec 2018 were enrolled as the development cohort to build a prediction model. The model was validated by internal and external validation. Patients between Jan 2019 and Dec 2019 were enrolled as the validation cohort. The adaptive least absolute shrinkage and selection operator (LASSO) was used to select the possible predictors. Receiving operating characteristic curve (ROC), calibration plots, and decision curve analysis (DCA) were used to evaluate the performance of the model. The performance of the Son score was also assessed.

**Results:**

LASSO regression demonstrated that age, sex, and CALs in the acute stage were predictors for CAA at one month. The area under the ROC (AUC) was 0.946 (95% confidence interval: 0.911–0.980) with a sensitivity of 92.5% and a specificity of 90.5%. The calibration curve and the DCA showed a favorable diagnostic performance. The internal and external validation proved the reliability of the prediction model. The AUC of our model and the Son score were 0.941 and 0.860, respectively (*P* < 0.001).

**Conclusion:**

Our prediction model for CAA at one month after disease onset in KD had an excellent predictive utility.

## Background

Kawasaki disease (KD) is one of the most common pediatric vasculitis all over the world. It is responsible for most acquired heart diseases due to its predilection for coronary arteries [1]. The incidence of coronary artery lesions (CALs) was reported to be approximately 25% in untreated patients [1]. Fortunately, nowadays these patients benefit greatly from the standard treatment regimen of intravenous immunoglobulin (IVIG) and aspirin in the acute stage. Most dilations were reported to resolve within 4 to 8 weeks of disease onset [2–4]. However, some coronary artery aneurysms (CAA), especially large or giant CAA, will persist for a long time, leading to a higher cardiac event risk in later life [3, 5, 6].

It was reported baseline CAA was associated with persistent CAA in Western countries whereas recent studies found that more than half of the coronary artery dilations recovered one month after disease onset, drawing a conclusion that CAA at one month after disease onset was of great significance and could act as a guide for late coronary outcomes [3, 4, 7–9]. Previously, Son and his colleagues established a risk model for CAA at 2 to 8 weeks in North America, which was identified to have a poor performance in the Japanese population [10, 11]. In China, the existing scoring systems for CAA were predictive of the occurrence of CAA in acute KD when the predictive model for CAA at one month after disease onset was still lacking [12, 13]. Therefore, the present study was designed to: (1) create a risk model for CAA prediction at one month with development and validation cohorts in an East China population. (2) identify the predictive utility of the Son score using data from this area. We aimed to facilitate the prediction of CAA at one month in clinical practice and further alleviate the unnecessary worries of the parents of patients with CAA which could possibly resolve over time.

## Materials and methods

### Patients

**Fig. 1:**
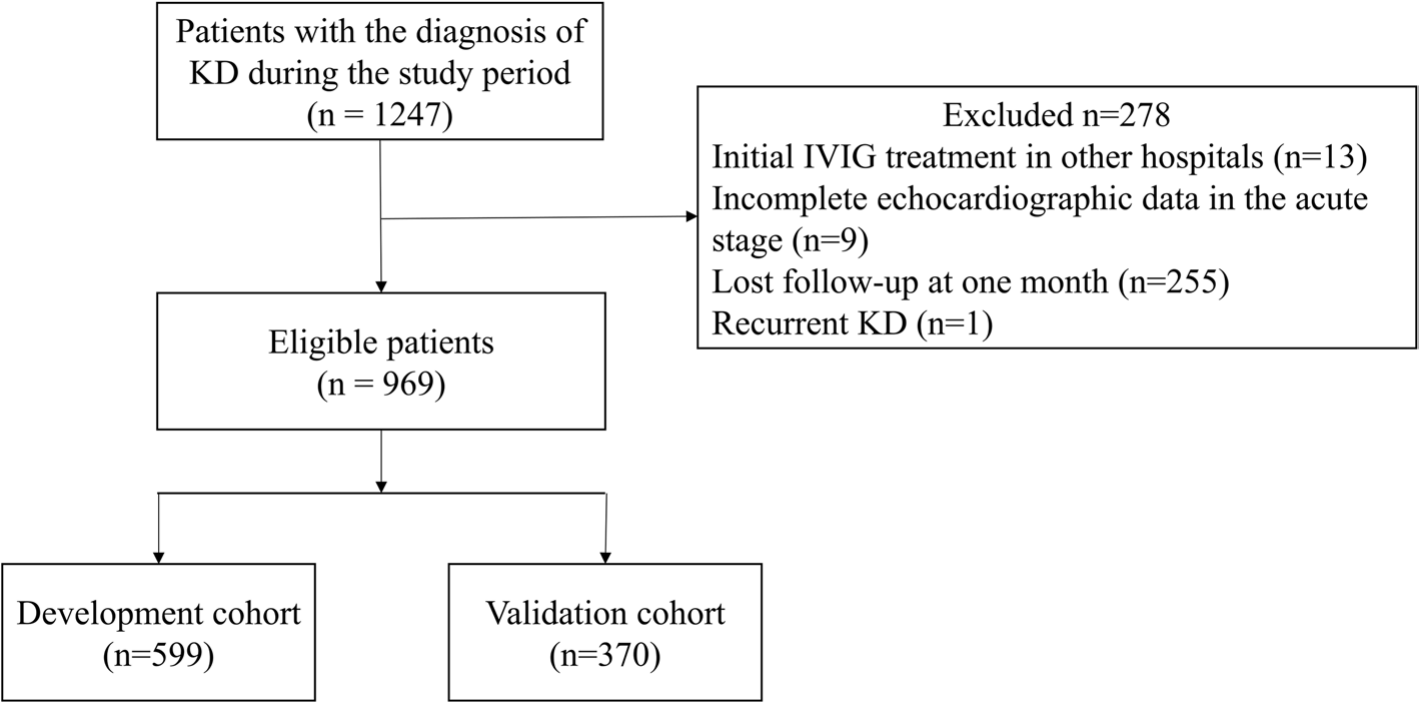
Study flow diagram

The study was conducted in Children’s Hospital of Soochow University. Children’s Hospital of Soochow University is a tertiary hospital that serves most patients in this area and some patients in surrounding areas. Patients with a diagnosis of KD between May 2017 and Dec 2018 were enrolled as the development cohort (DC), and patients between Jan 2019 and Dec 2019 were enrolled as the validation cohort (VC). Exclusion criteria were as follows: 1. Patients with initial IVIG in other hospitals, 2. Patients with incomplete echocardiographic data in the acute stage, 3. Patients with lost follow-up at one month after disease onset, 4. Patients with recurrent KD. As a result, a total of 599 patients were included in the DC and 370 patients were enrolled in the VC (Fig. 1).

The study was approved by the institutional review board of Children’s Hospital of Soochow University (No:2023cs031).

### Definitions

Diagnosis of KD was based on the 2017 AHA guideline [1]. A total of 2 g/kg IVIG in a single dose together with 30–50 mg/kg aspirin was administered in KD patients. For patients who were afebrile for 3–4 days, aspirin was then reduced to 3–5 mg/kg/day until the patients showed no evidence of CALs by 6 to 8 weeks after the onset of illness. Delayed IVIG treatment was defined as IVIG initiation after the 10^th^ day of illness. We calculated the Z scores of the left main coronary artery, left circumflex artery, left descending artery, and right coronary artery based on the method reported and CAA was defined by a Z score ≥ 2.5 [14]. No involvement of coronary arteries was defined as a Z score always < 2. Dilation was defined when the maximum Z score was between 2 and 2.5. A small aneurysm was defined when the maximum Z score was between 2.5 and 5.0. A medium aneurysm was defined when the maximum Z score was 5.0 and 10.0, and a giant aneurysm was defined when the maximum Z score was ≥ 10. IVIG resistance was defined as a persistent or recurrent fever ≥ 38.0 ℃ for more than 36 h.

According to the Son score, 2 points were assigned if the maximum Z score at baseline was ≥ 2, 1 point if age at fever onset was < 6 months, 1 point if Asian ethnicity was reported, and 1 point if baseline C-reactive protein (CRP) was ≥ 130 mg/L [10]. Because our study population were Chinese, all the patients had 1 point at baseline for ethnicity.

### Data collection

The medical data were prospectively collected. The body weight and height were collected to calculate the Z scores. Echocardiograms were performed before or within the first two days of IVIG treatment and were repeated before discharge and at four weeks (± one week). If the patients had severe complications, echocardiograms were repeated as appropriate. Medical data was collected which included demographics, incomplete KD, use of IVIG, days of IVIG initiation, response to IVIG treatment, total fever duration, and echocardiographic findings. Laboratory variable of CRP was also obtained to calculate the Son score. The putative predictors were chosen based on previous work that showed their potential prognostic value in CALs and on our clinical experience [4, 10, 12, 15].

### Statistical analyses

The sample size was derived based on the available data. Categorical variables were expressed as numbers and percentages. Continuous variables were shown as median with mean ± standard deviation (SD) or median with quartiles. In comparisons between two groups, Mann–Whitney U test, Student’s t test or chi-square test was used as appropriate. A penalised model of the adaptive least absolute shrinkage and selection operator (LASSO) was used to select the possible predictors because multicollinearity was present among CALs in the acute stage and other predictors. The performance of the model was evaluated by area under the receiving operating characteristic curve (AUC), calibration plots, and decision curve analysis (DCA). A nomogram risk prediction model was also established in the development cohort.

Model validation was done in two steps. First, we did an internal validation using a bootstrap resampling process to provide an unbiased estimate of model performance. Second, to assess external validity, the prediction accuracy of CAA at one month was determined on the validation cohort by computing the AUC, calibration plots, and DCA. Delong’s test was used to compare the AUC of two models. Minimum sample size required for the new model development was also calculated. All the data were analyzed using R for Windows (Version 4.0.4) with pmsampsize, glmnet, rms, rmda, caret, and pROC packages. Two-sided *P* < 0.05 was considered statistically significant.

## Results

### Patient characteristics

A total of 599 patients were enrolled in the DC including 372 males and 227 females. The minimum sample size calculated was 319 (R^2^ of the model was 0.2 and the number of variables was 8). The male-to-female ratio was 1.64. Fifty-three (8.8%) patients had CAA at one month. The comparisons of patients with and without CAA at one month are shown in Table 1. Patients with CAA at one month were younger, had later IVIG initiation, and had higher incidences of CALs in the acute stage. They also tended to have a longer fever duration, although the difference was not statistically significant (*P* = 0.054).

**Table 1:** Demographic and clinical comparisons between patients with and without coronary artery aneurysms (CAA) at one month

	Levels	Patients without CAA at one month	Patients with CAA at one month	*P*
Number		546	53	
Sex, n (%)	Females	213 (39.0)	14 (26.4)	0.098
	Males	333 (61.0)	39 (73.6)	
Age, months, (mean (SD))		28.04 (22.91)	16.92 (17.28)	0.001
Age, months, n (%)	< 12	125 (22.9)	28 (52.8)	< 0.001
	≥ 12 and <36	266 (48.7)	18 (34.0)	
	≥ 36 and < 60	98 (17.9)	5 (9.4)	
	≥ 60 and < 120	54 (9.9)	2 (3.8)	
	≥ 120	3 (0.5)	0 (0.0)	
Complete KD, n (%)	No	96 (17.6)	14 (26.4)	0.162
	Yes	450 (82.4)	39 (73.6)	
Use of IVIG, n (%)	No	5 ( 0.9)	0 (0.0)	1
	Yes	541 (99.1)	53 (100.0)	
Days of IVIG at initiation, (mean (SD))		6.90 (2.07)	8.11 (3.42)	< 0.001
Days of IVIG initiation, n (%)	< 5	32 (5.9)	1 (1.9)	< 0.001
	≥ 5 and< 8	339 (62.1)	28 (52.8)	
	≥ 8 and < 10	151 (27.7)	12 (22.6)	
	≥ 10	24 ( 4.4)	12 (22.6)	
Total fever duration, days, (mean (SD))		7.37 (2.35)	8.06 (3.42)	0.054
IVIG resistance, n (%)	No	514 (94.1)	50 (94.3)	1
	Yes	32 (5.9)	3 (5.7)	
CALs in acute stage, n (%)	No	492 (90.1)	10 (18.9)	< 0.001
	Yes	54 (9.9)	43 (81.1)	

*KD* Kawasaki disease, *IVIG* Intravenous immunoglobulin, *SD* Standard deviation, *CALs* Coronary artery leisions

Three hundred and seventy patients were enrolled in the VC and comparisons between DC and VC are shown in Table 2. The two cohorts differed in age, days of IVIG at initiation, and CALs in the acute stage. However, no difference was found regarding sex, complete KD, use of IVIG, total fever duration, and IVIG resistance.

**Table 2:** Baseline characteristics of all patients in the training cohort and validation cohort

	Levels	Development cohort	Validation cohort	*P*
Number		599	370	-
Sex, n (%)	Females	227 (37.9)	158 (42.7)	0.156
	Males	372 (62.1)	212 (57.3)	
Age, months, (mean (SD))		19.0 [11.0, 37.0]	26.0 [14.0, 45.0]	< 0.001
Age, months, n (%)	< 12	153 (25.5)	60 (16.2)	0.008
	≥ 12 and < 36	284 (47.4)	182 (49.2)	
	≥ 36 and < 60	103 (17.2)	84 (22.7)	
	≥ 60 and < 120	56 ( 9.3)	42 (11.4)	
	≥ 120	3 ( 0.5)	2 ( 0.5)	
Complete KD, n (%)	No	110 (18.4)	67 (18.1)	0.988
	Yes	489 (81.6)	303 (81.9)	
Use of IVIG, n (%)	No	5 ( 0.8)	8 ( 2.2)	0.145
	Yes	594 (99.2)	362 (97.8)	
Days of IVIG at initiation, (mean (SD))		7.00 (2.25)	7.35 (2.06)	0.019
Days of IVIG initiation, n (%)	< 5	33 ( 5.5)	14 ( 3.9)	0.201
	≥ 5 and< 8	367 (61.3)	205 (56.6)	
	≥ 8 and< 10	139 (23.2)	98 (27.1)	
	≥ 10	60 (10.0)	45 (12.4)	
Total fever duration, days, (mean (SD))		7.43 (2.47)	7.74 (2.26)	0.056
IVIG resistance, n (%)	No	564 (94.2)	340 (91.9)	0.216
	Yes	35 ( 5.8)	30 ( 8.1)	
CALs in the acute stage, n (%)	No	449 (75.0)	248 (67.0)	0.008
	Yes	150 (25.0)	122 (33.0)	

*KD* Kawasaki disease, *IVIG* Intravenous immunoglobulin, *SD* Standard deviation, *CALs* Coronary artery lesions

### Screening for predictive factors and prediction model development

**Fig. 2:**
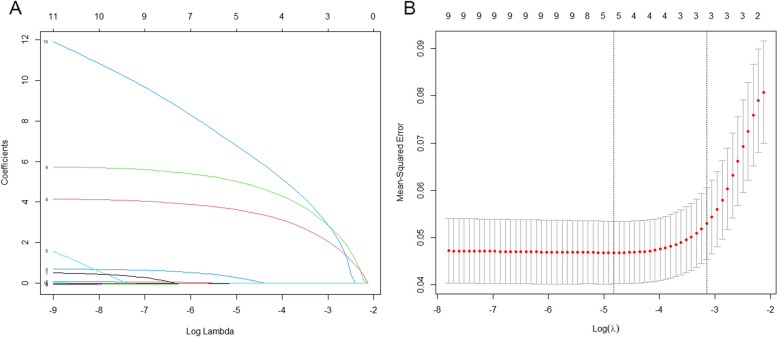
Variable selection by LASSO regression. **A** Three variables with nonezero coefficients were selected by optimal lambda. **B** A tenfold cross-validation was used in the LASSO regression. LASSO, least absolute shrinkage and selection operator

**Fig. 3:**
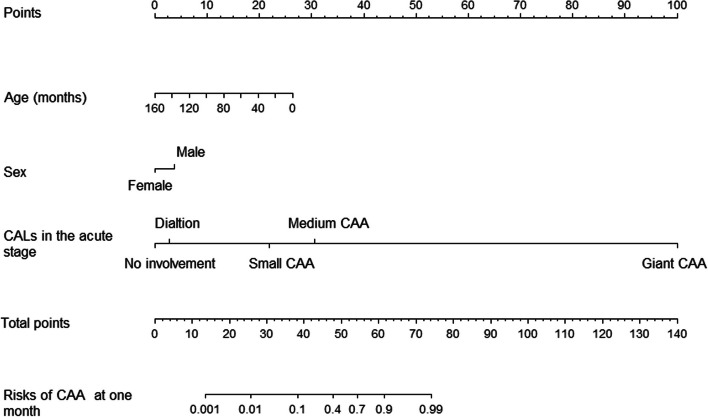
Nomogram for prediction of CAA at one month. CAA: coronary artery aneurysms

Predictors selected by LASSO regression were age, sex, and CALs in the acute stage (Fig. 2). These three predictors were integrated into the nomogram (Fig. 3). For each patient, a higher total point indicates a higher risk of CAA at one month. When the total points reached 75, an estimated incidence of CAA at one month was approximately 100%.

### Accuracy and net benefit of the model

**Fig. 4:**
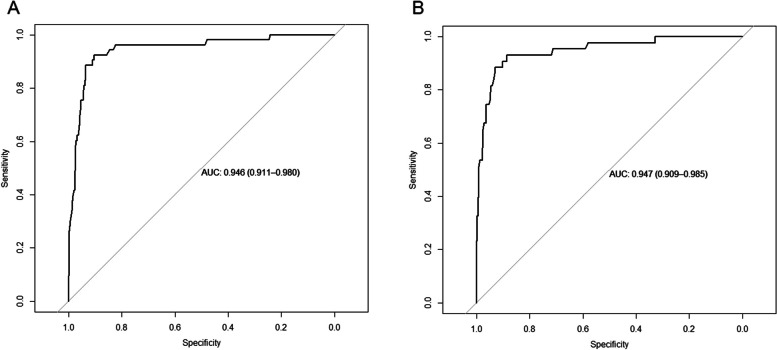
ROC curves of the prediction model. **A** Development cohort. **B** Validation cohort

**Fig. 5:**
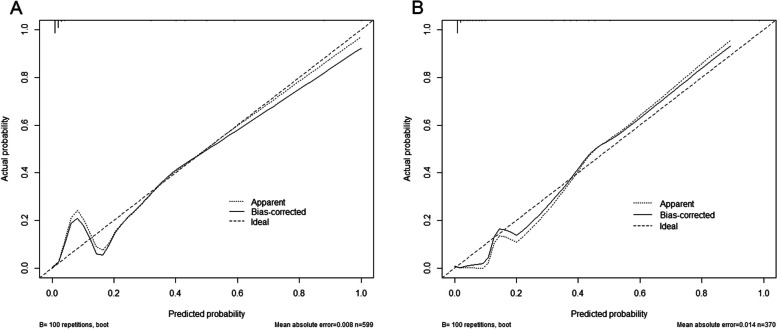
Calibration curve for the prediction model. **A** Development cohort. **B** Validation cohort

**Fig. 6:**
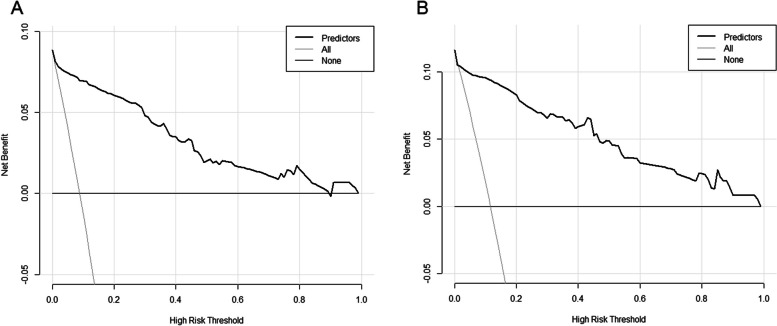
Decision curve analysis in the prediction model. **A** Development cohort. **B** Validation cohort

In the DC, the AUC of the model was 0.946 (95% confidence interval [CI]: 0.911–0.980) with a sensitivity of 92.5% and a specificity of 90.5% (Fig. 4A). Using ten-fold cross-validation, the AUC of the model was 0.930. The calibration curve was close to the ideal diagnostic line (Fig. 5A). The DCA showed significantly better net benefit in the predictive model (Fig. 6A). Another VC was used for external validation. The AUC was 0.947 (95% CI: 0.909–0.985) with a sensitivity of 93.0% and a specificity of 88.7% (Fig. 4B). Meanwhile, the model had good consistency, and the calibration curve of the validation cohort was also close to the ideal diagonal line (Fig. 5B). Moreover, the DCA showed a significant net benefit of the validation cohort (Fig. 6B).

### Evaluation of the son score

A total of 967 patients were enrolled to evaluate the performance of the Son score after excluding two patients with incomplete CRP. Two hundred and seventy-nine patients had a high-risk score, defined as ≥ 3 points, among whom 90 (32.3%) showed CAA at one month. At the cutoff ≥ 3 points, the sensitivity was 93.8%, the specificity was 78.3%, the positive predictive value was 32.3%, and the negative predictive value was 99.1%. The AUC for the Son score was 0.860 (95% CI: 0.832–0.888). DeLong's test for ROC curves of the Son score and our model showed the difference was statistically significant (AUC 0.860 vs. 0.941, *P* < 0.001).

## Discussion

In the present study, we developed a model for predicting CAA at one month in KD. The prediction model included age, sex, and CALs in the acute stage, which exhibited good performance in both the DC and VC. Besides, we evaluated the performance of the Son score, and found that although its sensitivity was quite high, it was no better than our prediction model in our cohort. These findings shed light on an early estimation of CAA at one month after KD onset.

Arteritis in KD model experienced three pathological processes, of which one month after disease onset fell into the subacute stage [1]. Pathologically, the coronary arteries were infiltrated with lymphocytes, plasma cells, and eosinophils without luminal myofibroblastic proliferation [1]. Clinically, patients were in the convalescent stage and routine follow-up was performed during this period [16]. Also, the Japanese Circulation Society guidelines of KD classified the severity of CALs based on the echocardiographic or coronary angiographic findings at one month from the onset [17]. Taken together, the coronary artery status at one month had a practical significance and an outsize role in clinical practice.

In previous multicenter studies in Japan and North America, an increased baseline Z score was recognized as a major risk factor for CALs at 1–2 months [8, 18, 19]. We also found that coronary artery status in the acute stage had a prominent predictive role in our model. When we classified acute CALs into five categories based on Z score, we found that dilations could possibly resolve and giant CAA would possibly persist, when the small and medium CAA were intermediate. Our results were in consistence with Ryusuke Ae’s [4]. He and his colleagues found that four-fifths of patients with dilatations improved at one month when aneurysms were less likely to improve over time. Indeed, larger aneurysms were prone to have a relatively poor prognosis, suffering from a series of cardiovascular events in later life [3, 5, 6].

Younger males were considered to be more vulnerable to KD. Moreover, younger age and males had been widely reported as risk factors for CALs in the acute stage, as well as at 1–2 months in a number of previous studies [8, 18–20]. Although the specific mechanism remained unclear, we speculated it might be related to the vulnerability of the immature coronary arteries in male infants caused by inflammation. Thus, pediatricians should pay more attention to these younger males with regard to a relatively unsatisfactory outcome. Unlike the results in the Post RAISE studies, IVIG resistance was not considered a risk factor in the present study [18, 19]. The AUC of IVIG resistance was 0.549 with a sensitivity of 15.6% and a specificity of 94.3%. The underlying reasons might be the divergence in different definitions of CALs, when we used Dallaire’s Z score equation and the Post RAISE studies used either Kobayashi’s Z score equation or the absolute coronary diameters to define CALs. Otherwise, there was no difference in CAAs between patients with and without IVIG resistance in our study, as was reported in another study which was carried out in Anhui, China [13].

When we compared our model with the Son score, we found that the latter was not more predictive than our model, although the Son score had a better performance in our cohort than in a Japanese one [11]. The reasons might lie in the different ethnic backgrounds of the patients enrolled and the unsatisfactory performance of CRP with an AUC of 0.557 (sensitivity: 27.1%, specificity: 84.3%) in our study. Actually, CRP was recognized as an independent risk factor of IVIG resistance rather than CALs in the acute stage in most studies.

The present study has several limitations. First, we excluded 255 patients who were lost in the one-month follow-up, which could lead to bias because most of the lost patients had less severe CALs in the acute stage and wouldn’t follow the doctor's advice after discharge. Second, the patients enrolled in our study were all Chinese, which would not guarantee the performance of the model in other nations. Moreover, a multicenter study is warranted in the future.

## Conclusion

Our prediction model for CAA at one month after disease onset in KD had an excellent predictive utility.

## Data Availability

The datasets used and/or analyzed during the current study are available from the corresponding author on reasonable request.

## Abbreviations

KD: Kawasaki disease
CALs: Coronary artery lesions
IVIG: Intravenous immunoglobulin
CAA: Coronary artery aneurysms
DC: Development cohort
VC: Validation cohort
CRP: C-reactive protein
SD: Standard deviation
LASSO: Adaptive least absolute shrinkage and selection operator
AUC: Area under the receiving operating characteristic curve
ROC: Receiving operating characteristic curve
DCA: Decision curve analysis
